# NEAT 1 knockdown enhances the sensitivity of human non-small-cell lung cancer cells to anlotinib

**DOI:** 10.18632/aging.203004

**Published:** 2021-05-12

**Authors:** Guoqing Gu, Chenxi Hu, Kaiyuan Hui, Ting Chen, Huiqin Zhang, Xiaodong Jiang

**Affiliations:** 1 Department of Oncology, The Affiliated Lianyungang Hospital of Xuzhou Medical University, Lianyungang, Jiangsu 222000, China

**Keywords:** NEAT 1, non-small cell lung cancer, anlotinib insensitiveness

## Abstract

Anlotinib treatment of non-small cell lung cancer (NSCLC) is hindered by drug insensitivity. Downregulation of long non-coding RNA (lncRNA) NEAT1 can suppress the proliferation and invasion by NSCLC cells. This study explored the role of the combination of anlotinib with NEAT1 knockdown on NSCLC progression. A549 and NCI-H1975 cells were used to evaluate the effect of anlotinib with NEAT 1 knockdown on NSCLC cells *in vitro*. The proliferation, invasion, migration, and apoptosis of NSCLC cells were evaluated with CCK-8 assays, EdU staining, Transwell assays, and flow cytometry. The antitumor effect of anlotinib with NEAT 1 knockdown was further explored in a mouse xenograft model. NEAT 1 knockdown enhanced the inhibitory effect of anlotinib on NSCLC cell proliferation, migration, and invasion. NEAT 1 knockdown also increased the pro-apoptotic and cytotoxic effects of anlotinib through downregulation of the Wnt/β-catenin signaling pathway. The inhibitory effect of anlotinib on tumor growth was boosted in the presence of NEAT 1 knockdown *in vivo*. NEAT 1 knockdown promoted NSCLC cell sensitivity to anlotinib *in vitro* and *in vivo*. Thus, combined treatment of anlotinib with NEAT 1 knockdown may provide a new combined therapeutic approach for NSCLC patients.

## INTRODUCTION

Lung cancer is the most frequently diagnosed cancer and a leading cause of cancer-related deaths worldwide [1, 2, 3]. Non-small cell lung cancer (NSCLC) accounts for 85% of lung cancer cases [4, 5]. For patients with NSCLC of stage I to IIIA, the five-year survival rate ranges from 14% to 49% [6]. However, for patients of stage IIIB/IV, the five-year survival rate is less than 5% [6]. Despite advancements in radiotherapy, immunotherapy, and chemotherapy on NSCLC patient outcomes, the prognosis remains unsatisfactory [6, 7]. Thus, improvements to current NSCLC therapeutic strategies are needed.

Anlotinib (1-[[4-(4-fluoro-2-methyl-1H-indol-5-yloxy)-6-methoxyquinolin-7-yl]oxy] methyl] cyclopropanamine dihydrochloride) is an orally administered multi-targeting tyrosine kinase inhibitor (TKI) that was recently developed by Chia-tai Tianqing Pharmaceutical Co., Ltd. in China [8]. The prime targets of anlotinib are receptor tyrosine kinases including vascular endothelial growth factor receptor (VEGFR), platelet-derived growth factor receptors (PDGFR), fibroblast growth factor receptor (FGFR), and c-kit [8–10]. As a multitarget TKI, anlotinib functions by inhibiting tumor angiogenesis and proliferation [11]. In May 2018, The China National Medical Products Administration (NMPA) approved anlotinib use as a third-line or further treatment for patients with NSCLC [12]. Anlotinib has demonstrated its efficacy in extending the progression-free survival (PFS) and overall survival (OS) of patients with advanced refractory NSCLC [13]. However, the occurrence of drug insensitiveness impeded its anti-tumor effectiveness [14].

Long non-coding RNA (lncRNA) are a group of non-protein coding RNAs that are more than 200 nucleotides in length [15]. LncRNAs regulate transcription at multiple levels including transcriptional, translational, and post-translational [15, 16]. The aberrant expression of lncRNAs has recently emerged as biomarkers, prognostic factors, and therapeutic targets for cancers [17]. In a previous study by Zhao et al., the knockdown of long noncoding RNA nuclear paraspeckle assembly transcript 1 (NEAT1) inhibited NSCLC progression *in vitro* [18]. However, the role of the combined NEAT1 and anlotinib treatment on NSCLC progression is not well known. In this study, we explored the effect of anlotinib and NEAT1 knockdown on NSCLC *in vitro* and *in vivo*.

## MATERIALS AND METHODS

### Reagents

Anlotinib hydrochloride (purity >99%) was manufactured by Chia Tai Tianqing Pharmaceutical Co, Ltd (Nanjing, China) and dissolved in physiological saline to various concentrations. Primary and secondary antibodies against VEGFR2, p-VEGFR2, and β-actin were purchased from Abcam (Cambridge, MA, USA). β-catenin and c-Myc were provided by Cell Signaling Technology (Danvers, MA, USA). Matrigel was obtained from BD Bioscience (Pasadena, CA, USA). Tris-buffered saline with 0.1% Tween-20 (TBST) was purchased from Sigma Aldrich.

### Cell culture of A549 and NCI-H1975

A549 and NCI-H1975 cells were acquired from ATCC (American Type Culture Collection, Manassas, VA, USA) and cultured in RPMI 1640 (Thermo Fisher Scientific, Waltham, MA, USA) supplemented with 100 U/ml penicillin, 0.1 mg/ml streptomycin, and 10% FBS. The cells were maintained in a humidified incubator at 37°C with 5% CO_2_.

### Knockdown of NEAT1

The siRNAs (10 nM) and negative control (siRNA-NC) for NEAT1 were acquired from Shanghai Genepharma. A549 and NCI-H1975 cells were plated onto 6-well plates and cultured overnight. siRNA1, siRNA2, or siRNA3 were transfected into the cells using Lipofectamine 3000 for 24 h (Invitrogen, Carlsbad, CA, USA). The siRNA sequences were follows: siRNA1, 5′-CCGTGGTGTGTGTTGTGGAATCTGT-3′; siRNA2, 5′-CGTGGTGTGTGTTGTGGAATCTGTG-3′; siRNA3, 5′-TGGTGTGTGTTGTGGAATCTGTGTT-3′; siRNA-NC: 5′-CCGTGTGTGTGGTGTAGTACGTTGT-3′.

### Overexpression of NEAT1

NEAT1 pcDNA3.1 (10 nM) and negative control (pcDNA3.1-ctrl) for NEAT1 were acquired from Shanghai Genepharma. NCI-H1975 cells were plated onto 6-well plates and cultured overnight. NEAT1 pcDNA3.1 or pcDNA3.1-ctrl was transfected into the cells using Lipofectamine 3000 for 24 h (Invitrogen, Carlsbad, CA, USA).

### Quantitative real-time PCR (RT-qPCR)

Total RNA was extracted from A549 and NCI-H1975 cells using TRIzol (Thermo Fisher Scientific). The extracted RNA was reverse-transcribed into cDNA using the PrimeScript First Strand cDNA synthesis kit (Takara, Dalian, China). Real time qPCR was performed using QuantiTect SYBR Green PCR Kits (Qiagen, Hilden, Germany) on an ABI Prism 7500 Sequence Detection System (Perkin-Elmer Inc., Waltham, MA, USA). Relative gene expression was normalized to ACTIN. All procedures followed the manufacture’s protocols. The primer sequences were NEAT1: sense, 5′-AGTGATGTGGAGTTAAGGCGC-3′; antisense, 5′-CGGGCTTACCAGATGACCAG-3′. ACTIN: sense, 5′-GTCCACCGCAAATGCTTCTA-3′; antisense, 5′-TGCTGTCACCTTCACCGTTC-3′.

### Cell counting kit-8 (CCK-8) assay

The CCK-8 kit (Dojindo, Kumamoto, Japan) was used to measure cell viability. A549 and NCI-H1975 cells from each group were seeded onto 96-well plates at a density of 3,500 cells/well and cultured overnight. After the indicated treatments, the cells were washed twice with PBS. Then, cells were incubated with the CCK-8 reagent at 37°C for 60 min following the manufacturer’s procedures. An optical density at 450 nm indicated cell viability and was measured by a microplate reader.

### Combination studies

The drug combination study was conducted by calculating the combination index (CI) using the Chou–Talalay method [19]. A549 and NCI-H1975 cells were exposed to anlotinib at 0, 10, 20, 30, or 40 μM with or without supplementing with NEAT1 siRNA3 (10 nM). The CI value for the combination of anlotinib and NEAT1 siRNA3 was calculated as previously described using the following formula: CI = DA/ICx,A + DB/ICx,B [20].

### 5-ethynyl-2′-deoxyuridine (EdU) fluorescence staining

EdU staining was employed using the Cell-Light EdU DNA cell proliferation kit (RiboBio, Guangzhou, China) to determine the proliferation of NCI-H1975 and A549 cells after the indicated treatments. All experimental steps were conducted according to manufacturer’s instructions. The frequency of EdU positive cells was calculated from three random fields.

### Cell invasion assay

Cell invasion assays were conducted with Transwell chambers (Corning, NY, USA) coated with Matrigel (BD Biosciences, Franklin Lakes, NJ, USA). After 24 h of transfection, A549 and NCI-H1975 cells were harvested, resuspended in 200 μL serum-free medium, and seeded onto the upper chamber at density of (3 × 10^4^/well). Then, anlotinib (20 μM) was used to treat the cells for 24 h. Cell culture medium supplemented with 10% FBS was added to the lower chamber to stimulate cell invasion. After 24 h of incubation, invaded cells were fixed with 4% paraformaldehyde. The invaded cells were stained with crystal violet (0.1%) for 15 min. Images were captured under an Olympus microscope, and the invaded cells were counted from three microscopic fields.

### Wound healing assay

After 24 h of transfection, A549 and NCI-H1975 cells were seeded onto 6-well plates and cultured until the cells reached 90% confluence. A sterile 100 μL pipette tip was used to scrape the wound. The cells were treated with culture medium supplemented with anlotinib (20 μM). After 24 h of incubation, the cells were fixed with 4% paraformaldehyde. The migration rate was measured based on the migration distances.

### Apoptosis assay

Apoptotic cells were determined by flow cytometry (FACScan, BD Biosciences) after the cells were doubled stained with Annexin V and propidium iodide (PI). The Annexin V-FITC Apoptosis Detection Kit (Thermo Fisher Scientific) was used for the apoptosis assay as per the manufacturer’s protocols.

### Western blot assay

Cells from each group were lysed with RIPA buffer (Beyotime Biotechnology, Shanghai, China). The concentration of total protein was determined using a BCA kit (Pierce Biotechnology, Rockford, IL, USA). Total protein of 30 μg was resolved by SDS-PAGE, transferred onto a PVDF membrane, blocked with 5% non-fat milk, and immunoblotted with antibodies. TBST was used as the washing buffer. Immunoblots were visualized using ECL detection kits (Merck Millipore, Billerica, MA, USA).

### Mouse xenograft models

Four-week-old BALB/c nude mice were acquired from Vital River (Beijing, China), housed in a standard animal laboratory, and allowed free access to water and food. All procedures involving animals were performed in accordance with the NIH Guide for the Care and Use of Laboratory Animals. The protocol was approved by the ethics committee for laboratory animal care and use of the Affiliated Lianyungang Hospital of Xuzhou Medical University. To induce tumor formation, 5 × 10^6^ A549 cells were injected subcutaneously into the right flank of each mouse. When the tumor volume reached 180 mm^3^, the mice were randomly separated into three groups (*n* = 6 per group). Anlotinib or the combination of anlotinib and NEAT1 siRNA3 were used to treat the mice. Anlotinib was administrated at 6 mg/kg/day for 14 days. NEAT1 siRNA3 (50 nM) was injected into the tumor twice per week. Mice injected with physiological saline were used as controls. All mice were sacrificed at week 4 after treatment. Tumors were harvested and weighed immediately after sacrifice. Tumor volumes were calculated using the following standard formula: length × width^2^/2.

### Statistical analysis

All experimental data are presented as the mean ± SD. At least three replicates were performed for all experiments. Significant differences between groups were evaluated by one-way ANOVA followed by a post hoc Tukey's test using GraphPad Prism 8 software.

## RESULTS

### NEAT1 knockdown increases the inhibitory effect of anlotinib on cell viability

**Figure 1:**
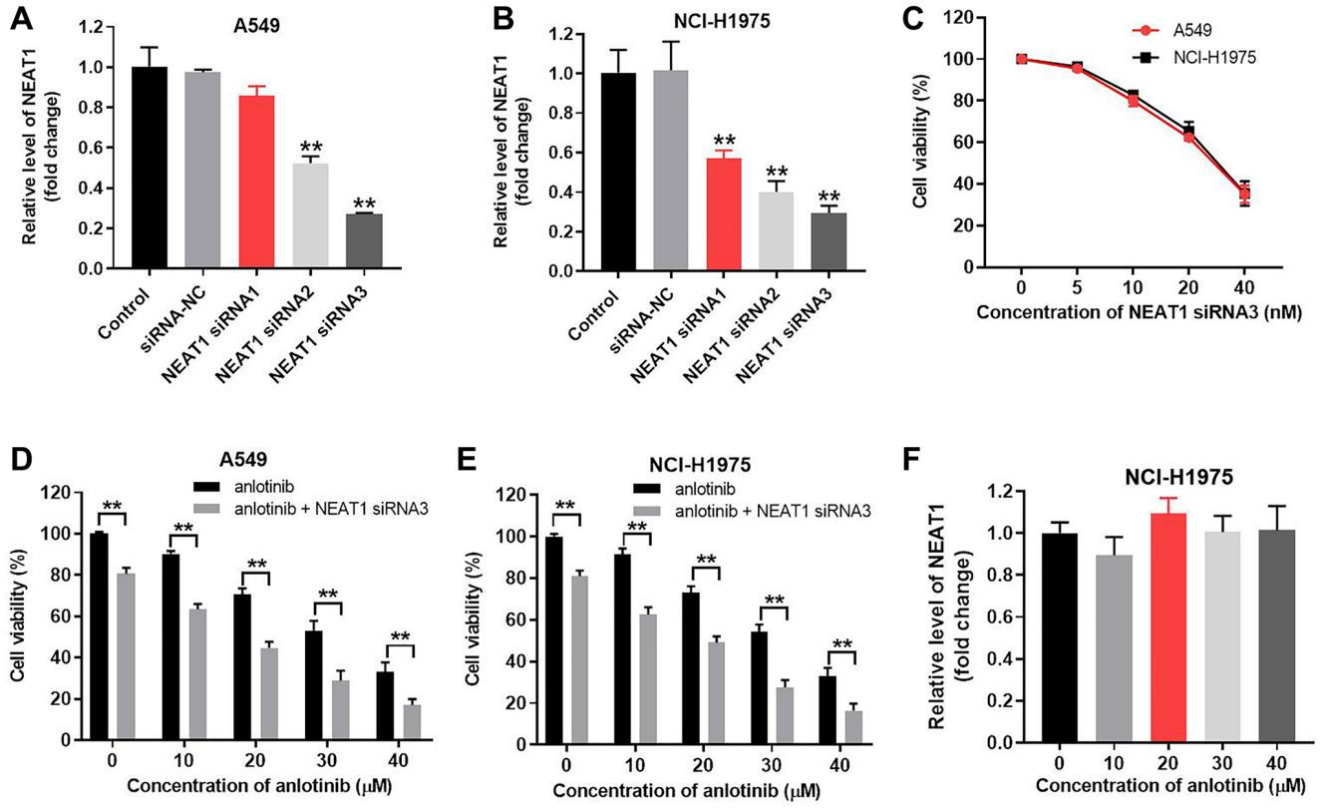
**NEAT1 knockdown increases the inhibitory effect of anlotinib on A549 and NCI-H1975 cell viability.** A549 and NCI-H1975 cells were transfected with siRNA-NC and NEAT1 siRNA 1, siRNA 2, or siRNA 3 for 24 h. NEAT1 levels were measured by RT-qPCR. (**A**) NEAT 1 levels in A549 cells and (**B**) NCI-H1975 cells. Cells were transfected with NEAT1 siRNA 3 (0, 5, 10, 20, 40 nM) for 24 h. (**C**) CCK-8 assays evaluated cell viability. (**D**–**E**) A549 and NCI-H1975 cells were treated with anlotinib or the combination of anlotinib and NEAT1 siRNA 3 for 24 h. (**F**) NCI-H1975 cells were treated with anlotinib (0, 10, 30, 40 μM) for 24 h and the level of NEAT1 was detected with RT-qPCR. ^**^P < 0.01 compared with the control group.

NEAT1 siRNA1, siRNA2, or siRNA3 were transfected into A549 and NCI-H1975 cells to knockdown NEAT1. The knockdown efficacy of each siRNA was detected with RT-qPCR after 24 h of transfection. NEAT1 siRNA3 achieved the best knockdown efficacy in A549 and NCI-H1975 cells (Figure 1A, 1B). Therefore, NEAT1 siRNA3 was utilized for knockdown experiments. Next, NEAT1 siRNA3 (0, 5, 10, 20, 40 nM) were transfected into A549 and NCI-H1975 cells. The influence of NEAT1 siRNA3 on the cell viability of NSCLC cells was evaluated by CCK-8 assays. As shown in Figure 1C and Supplementary Figure 1A, NEAT1 siRNA3 repressed the viability of A549, NCI-H1975 and BEAS-2B cells. A 10 nM concentration of NEAT1 siRNA3 induced a moderate cell viability decline and was subsequently used to treat NSCLC cells together with anlotinib. The effect of NEAT1 siRNA3 combined with anlotinib (from 0 to 40 μM) on cell viability was detected by CCK-8 assays. As indicated in Figure 1D and 1E, anlotinib alone suppressed the viability of A549 and NCI-H1975 cells. NEAT1 siRNA3 further enhanced the inhibitory effect of anlotinib on NSCLC cell viability. However, BEAS-2B cell viability of anlotinib group and anlotinib + NEAT1 siRNA3 group exhibited no significant difference (Supplementary Figure 1B). NEAT1 siRNA2 exhibited similar effects on NCI-H1975 cell viability (Supplementary Figure 1A). In addition, anlotinib had no effect on the expression of NEAT1 in NCI-H1975 cells (Figure 1F).

The IC50 value of anlotinib was 23.73 μM and 24.72 μM in A549 and NCI-H1975 cells, respectively. When combined with NEAT1 siRNA3 (10 nM), the IC50 value was decreased to 8.32 μM and 8.55 μM, respectively. Moreover, the CI value of the combination of anlotinib and NEAT1 siRNA3 was 0.67 in A549 cells and 0.65 in NCI-H1975 cells (Table 1). The CI values were > 0.6 but < 0.8, which indicates a moderate synergism effect [20]. These results suggest that combining anlotinib with NEAT1 siRNA3 synergistically inhibits the viability of A549 and NCI-H1975 cells. In contrast, overexpression of NEAT1 reversed the inhibitory effect of anlotinib on NCI-H1975 cell viability (Supplementary Figure 3A and 3B).

**Table 1:** Evaluation of combination of anlotinib with NEAT1 siRNA3 in A549 and NCI-H1975 cells (48 h treatment).

**Drug combination**	**A549 cells**		**NCI-H1975 cells**	
	**IC 50 value**	**CI values**	**IC 50 value**	**CI values**
Anlotinib (range 0 from 40 μM)	IC50 = 23.73 μM	–	IC50 = 24.72 μM	–
Anlotinib + NEAT1 siRNA3 (10 nM)	IC50 = 8.32 μM	0.67	IC50 = 8.55 μM	0.65

### NEAT1 knockdown enhances the inhibitory effect of anlotinib on cell proliferation

**Figure 2:**
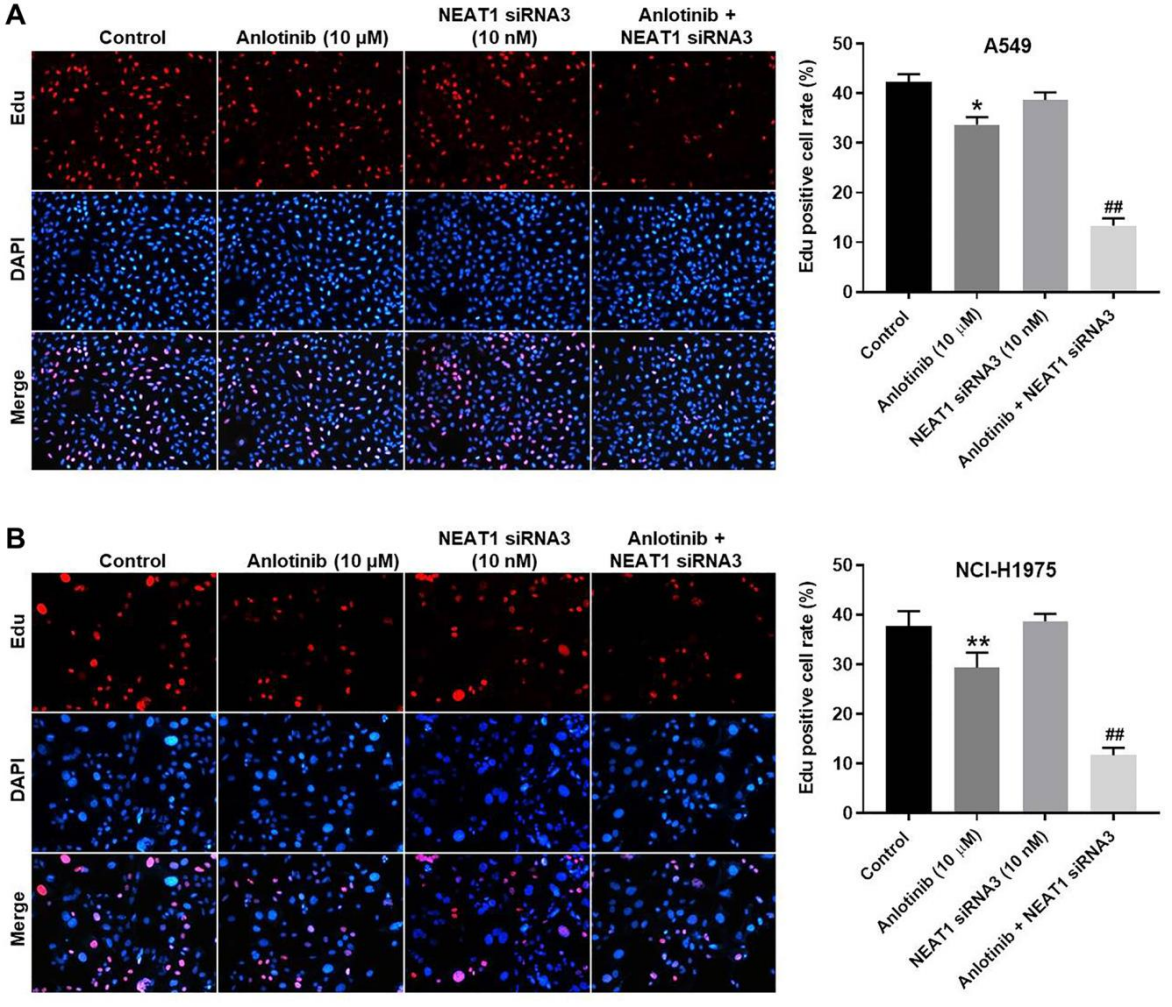
**NEAT1 knockdown enhances the inhibitory effect of anlotinib on the proliferation of A549 and NCI-H1975 cells.** Cells were treated with anlotinib (10 μM), NEAT1 siRNA 3 (10 nM), or the combination of anlotinib (10 μM) and NEAT1 siRNA 3 (10 nM) for 24 h. EdU staining was used to detect cell proliferation. (**A**) Cell proliferation in A549 cells and (**B**) NCI-H1975 cells. ^*^P < 0.05, ^**^P < 0.01 compared with the control group. ^##^*P* < 0.01, compared with the anlotinib group.

EdU staining was used to determine the effect of NEAT1 siRNA3 in combination with anlotinib on cell proliferation. Anlotinib inhibited proliferation in A549 and NCI-H1975 cells (Figure 2A, 2B). Sole treatment with NEAT1 siRNA3 (10 nM) had no significant effect on the proliferation (Figure 2A, 2B). However, the combined treatment of NEAT1 siRNA3 with anlotinib remarkably enhanced the anti-proliferation effect of anlotinib in A549 and NCI-H1975 cells (Figure 2A, 2B).

### NEAT1 knockdown increases the anti-invasion and anti-migration effect of anlotinib in A549 and NCI-H1975 cells

**Figure 3:**
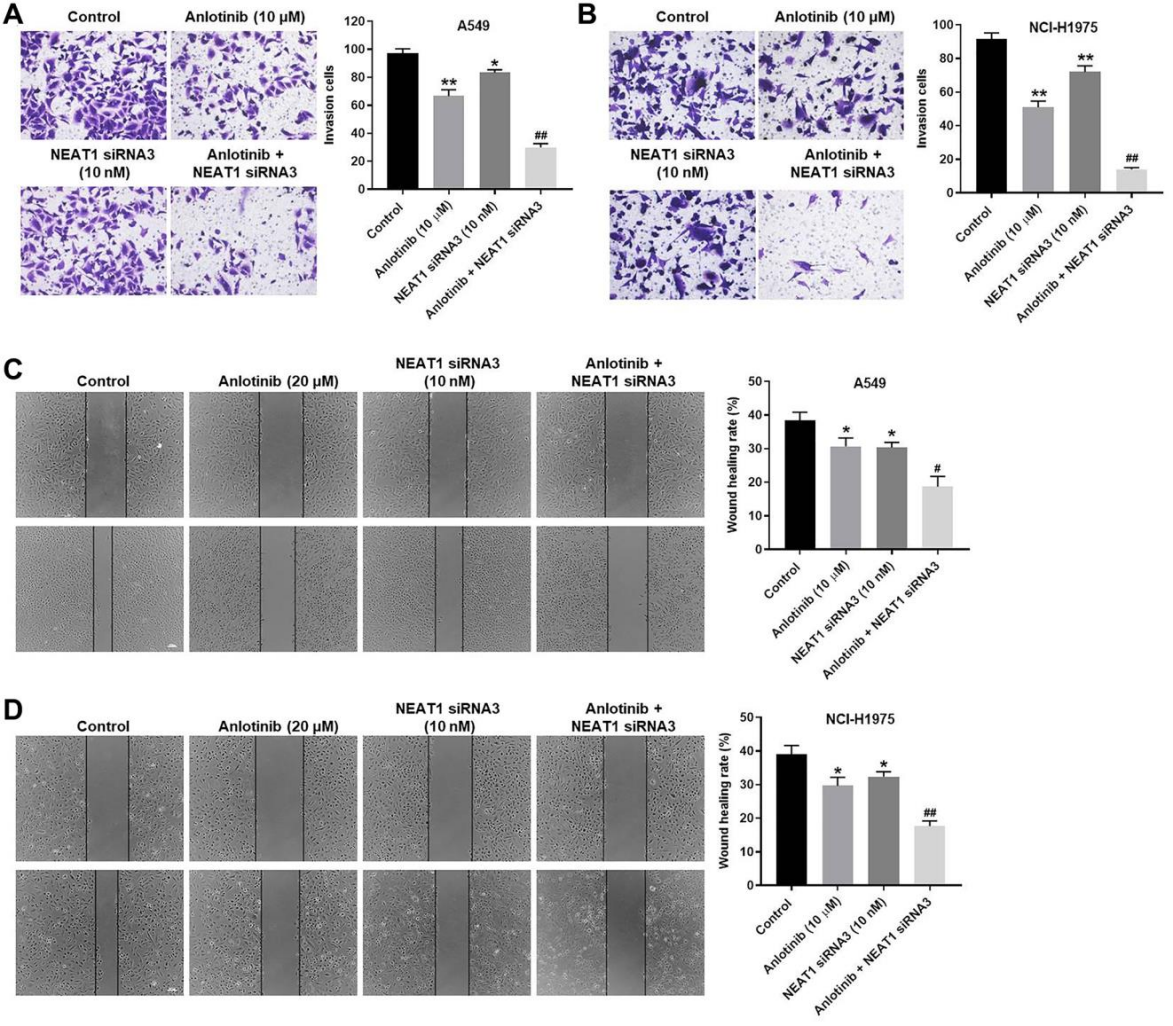
**NEAT1 knockdown increases the anti-invasion and anti-migration effect of anlotinib on A549 and NCI-H1975 cells.** Cells were treated with anlotinib (10 μM), NEAT1 siRNA 3 (10 nM), or the combination treatment for 24 h. (**A**–**B**) Cell invasion assays were conducted on A549 and NCI-H1975 cells. Migrated cells were quantified. (**C**–**D**) Wound healing assays were performed in A549 and NCI-H1975 cells, and the wound healing rate was quantified. ^*^P < 0.05, ^**^P < 0.01 compared with the control group. ^#^*P* < 0.05, ^##^*P* < 0.01, compared with the anlotinib group.

To explore the effect of NEAT1 siRNA3 and anlotinib on the invasion and migration of A549 and NCI-H1975 cells, cell invasion and wound healing assays were conducted, respectively. Anlotinib or NEAT1 siRNA3 suppressed the invasion of A549 and NCI-H1975 cells (Figure 3A, 3B). The inhibitory effect of anlotinib on cell invasion was improved by combined treatment with NEAT1 siRNA3 or with NEAT1 siRNA2 (Figure 3A, 3B and Supplementary Figure 1B). Anlotinib or NEAT1 siRNA3 repressed the migration of A549 and NCI-H1975 cells (Figure 3C, 3D). The combined treatment exhibited a greater inhibitory effect on cell migration compared with anlotinib alone. These results demonstrate that NEAT1 knockdown increases the anti-invasion and anti-migration effect of anlotinib in A549 and NCI-H1975 cells.

### NEAT1 knockdown enhances the anti-apoptotic effect of anlotinib

**Figure 4:**
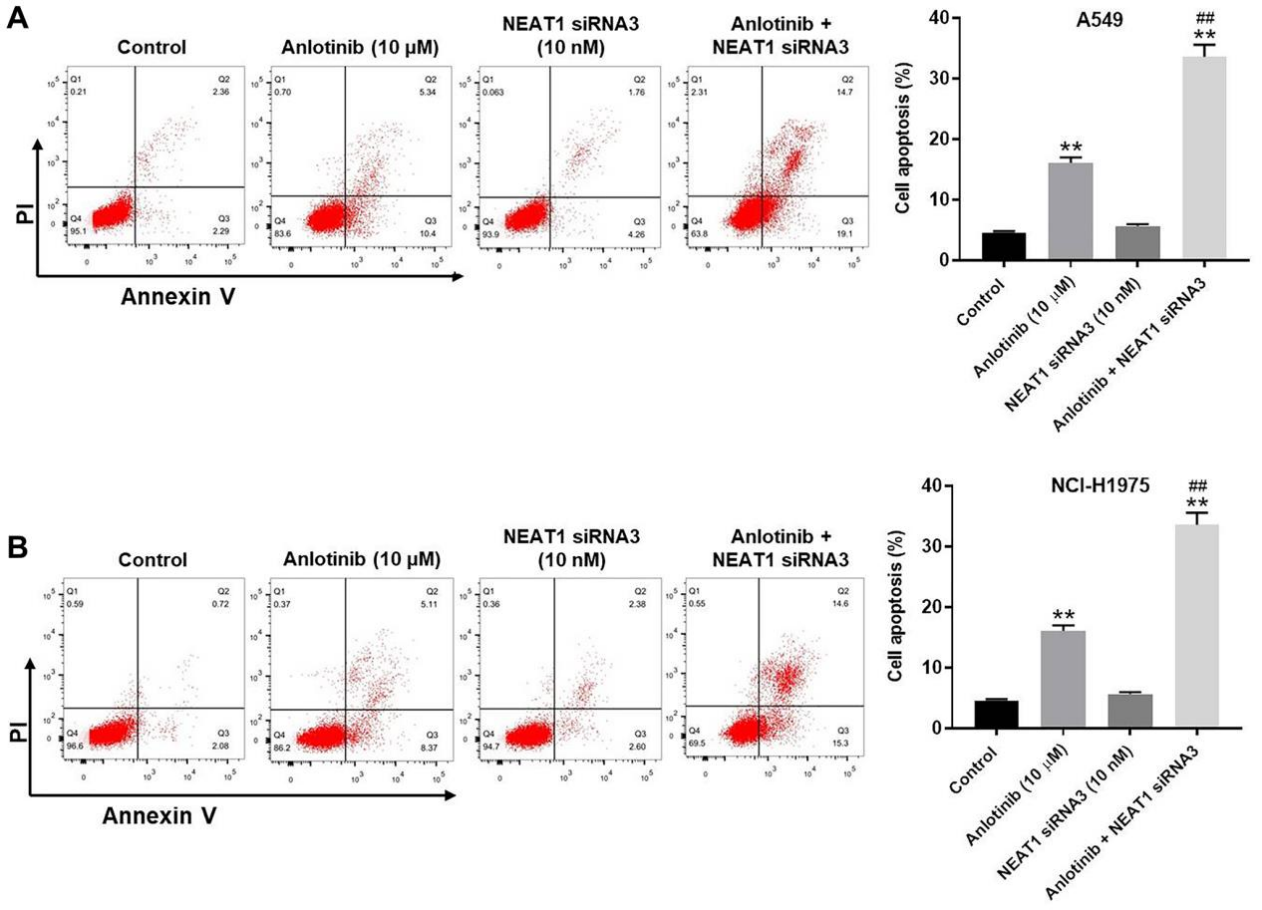
**NEAT1 knockdown enhances the anti-apoptotic effect of anlotinib in A549 and NCI-H1975 cells.** After 24 h of the indicated treatments, Annexin V and PI double staining was performed to detect cell apoptosis and the rate was quantified. (**A**) Cell apoptosis assays for A549 cells and (**B**) NCI-H1975 cells. ^**^P < 0.01 compared with the control group. ^##^*P* < 0.01, compared with the anlotinib group.

We examined the effect of NEAT1 siRNA3 and anlotinib on cell apoptosis through Annexin V/PI staining. NEAT1 siRNA3 had little effect on apoptosis in the A549 and NCI-H1975 cells (Figure 4A, 4B). In contrast, anlotinib increased cell apoptosis, which was enhanced by the presence of NEAT1 siRNA3 (Figure 4A, 4B). In addition, Restoration of NEAT1 expression could reverse the effect of anlotinib on cell viability (Supplementary Figure 2A, 2B). These results illustrate that NEAT1 knockdown improves the anti-apoptotic effect of anlotinib in A549 and NCI-H1975 cells.

### NEAT1 siRNA3 facilitates the cytotoxic effect of anlotinib through downregulation of the Wnt/β-catenin signaling pathway

**Figure 5:**
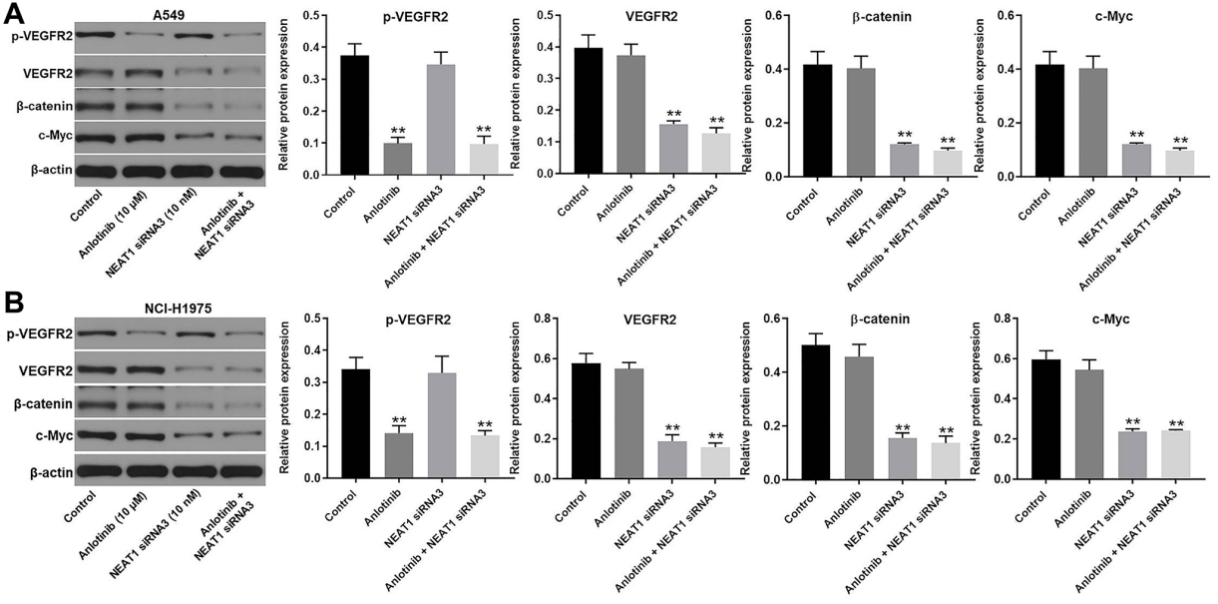
**NEAT1 siRNA3 facilitated the cytotoxic effect of anlotinib through downregulation of Wnt/β-catenin signaling pathway.** The expression of p-VEGFR2, VEGFR2, β-catenin, and c-Myc were evaluated by western blot. β-actin was used as an inner control. (**A**) The expression of *p*-VEGFR2, VEGFR2, β-catenin, and c-Myc in A549 cells and (**B**) NCI-H1975 cells. ^**^P < 0.01 compared with the control group.

Western blot was utilized to explore the mechanism of the combination treatment in A549 and NCI-H1975 cells. NEAT1 can act as an oncogenic IncRNA in NSCLC through modulating the WNT/β-catenin signaling pathway [21]. Thus, the expression of β-catenin and c-Myc were detected. Since anlotinib is a VEGFR2 inhibitor, the expression of VEGFR2 and p-VEGFR2 were also detected by western blot [9]. As shown in Figure 5A and 5B, anlotinib inhibited the expression of p-VEGFR2 and had no influence on the expression of VEGFR2, β-catenin, or c-Myc in A549 and NCI-H1975 cells (Figure 5A, 5B). NEAT siRNA3 alone had no effect on p-VEGFR2 expression but suppressed the expression of VEGFR2, β-catenin, and c-Myc in A549 and NCI-H1975 cells (Figure 5A, 5B). In addition, the combination of NEAT siRNA3 with anlotinib repressed the expression of p-VEGFR2, VEGFR2, β-catenin, and c-Myc in A549 and NCI-H1975 cells (Figure 5A, 5B). The anti-proliferative effect of the combination treatment in normal lung epithelial line (BEAS-2B) was tested as well. The results indicated only high concentration of NEAT1 siRNA (40 nM) and anlotinib (40 μM) had some cytotoxicity. Routine concentration of NEAT1 siRNA (10 nM) and anlotinib (10 μM) had no cytotoxicity on BEAS-2B cells (Supplementary Figure 3A–3B). These results demonstrate that NEAT1 siRNA3 enhances the sensitivity of NSCLC cells to anlotinib by regulating the WNT/β-catenin signaling pathway.

### NEAT 1 knockdown enhances the anti-tumor effect of anlotinib *in vivo*

**Figure 6:**
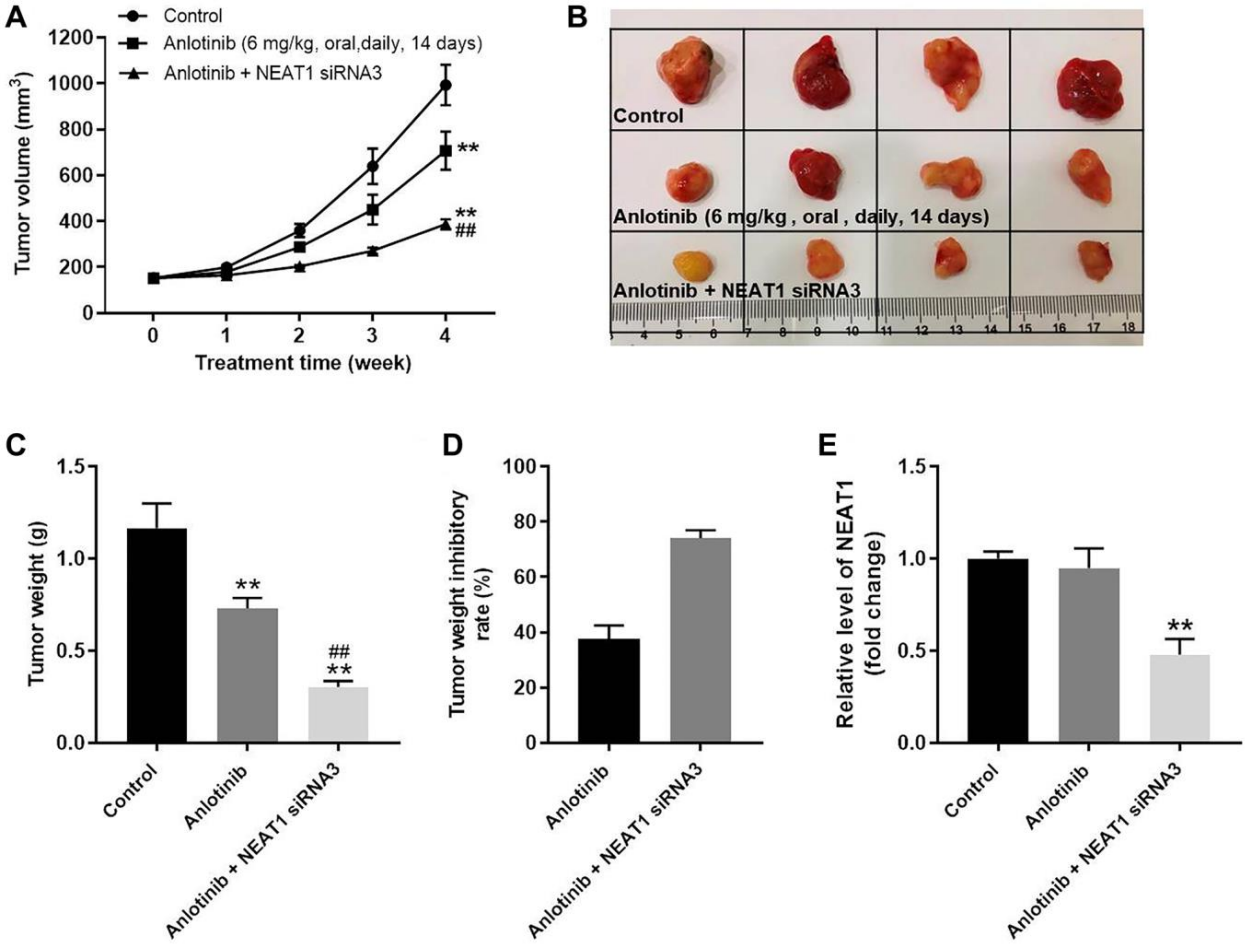
**NEAT 1 knockdown enhances the anti-tumor effect of anlotinib *in vivo*.** To form subcutaneous tumors, 5 × 10^6^ A549 cells were injected subcutaneously into the right flank of each mouse. After the tumor volume reached 180 mm^3^, the mice were treated with anlotinib or anlotinib+NEAT1 siRNA3. (**A**) Tumor volume was measured at week 0, 1, 2, 3, and 4. (**B**) Mice were sacrificed at week 4 after treatment. Tumors were harvested and imaged. (**C**) Tumor weight was evaluated. (**D**) Calculated tumor weight inhibitory rates. (**E**) The expression of NEAT1 in tumor tissues was detected with RT-qPCR. ^**^P < 0.01 compared with the control group. ^##^*P* < 0.01, compared with the anlotinib group.

The effect of NEAT1 siRNA3 combined with anlotinib on tumor growth was investigated in a mouse xenograft model. As shown in Figure 6A–6C, sole treatment with anlotinib inhibited the increase of tumor volume and weight compared to the control group. Moreover, the combined treatment of NEAT1 siRNA3 with anlotinib remarkably decreased the tumor volume and weight compared to sole anlotinib treatment (Figure 6A–6C). The tumor weight after anlotinib treatment was improved from approximately 40% to near 80% after combined therapy with NEAT1 siRNA3 (Figure 6D). In addition, NEAT1 siRNA3 could effectively inhibit the level of NEAT1 in tumor tissues (Figure 6E). These findings suggest that NEAT 1 knockdown enhances the anti-tumor effect of anlotinib *in vivo*.

## DISCUSSION

Anlotinib is used as a third-line or further treatment for advanced refractory NSCLC due to its efficacy in extending PFS and OS [12, 13]. However, drug insensitiveness obstructs its anti-tumor effectiveness. Knockdown of NEAT1 can inhibit NSCLC progression *in vitro*, but the role of NEAT1 in enhancing the sensitivity to anlotinib remains unexplored [18]. Our findings showed for the first time that combined treatment of NEAT1 and anlotinib inhibited the progression of NSCLC.

In addition, it was previously reported that NEAT1 affects NSCLC cells by regulating Wnt/β-catenin signaling [21]. In particular, the downregulation of NEAT1 repressed the activity of Wnt/β-catenin signaling pathway, which suppressed the proliferation, migration, and invasion of NSCLC cells [21]. Our findings demonstrated that NEAT1 siRNA3 enhanced the sensitivity of NSCLC cells to anlotinib by inhibiting the Wnt/β-catenin signaling pathway. Thus, our findings further validate the involvement of Wnt/β-catenin signaling pathway in NEAT1 on NSCLC progression.

However, the regulatory mechanism of NEAT1 in combination with anlotinib on NSCLC cells has not been fully revealed. Yu et al. reported that Krüppel-like factor 3 (KLF3) was associated with the role of NEAT1 in regulating the proliferation, apoptosis, and invasion of NSCLC cells [22]. According to the findings of Yu et al., LncRNA NEAT1 sponges microRNA(miR)-1224. miR-1224 then binds to 3′UTR of Krüppel-like factor 3 (KLF3), affecting the proliferation, apoptosis, and invasion of A549 cells [22]. Kong et al. reported that NEAT1 promoted NSCLC progression through the miR-101-3p/SOX9/Wnt/β-Catenin signaling pathway [23]. Taken together, these mechanisms of NEAT1 on NSCLC progression need to be further investigated when NEAT1 is used in combination with anlotinib.

In conclusion, we demonstrated that NEAT 1 knockdown promotes the sensitivity of NSCLC cells to anlotinib through downregulation of the Wnt/β-catenin signaling pathway. The combined treatment of anlotinib with NEAT 1 knockdown provides novel insights on developing a combined therapeutic approach against anlotinib insensitiveness in NSCLC patients.

## Supplementary Materials

Supplementary Figures
