# Supplementary material for: NEAT 1 knockdown enhances the sensitivity of human non-small-cell lung cancer cells to anlotinib
Source: Aging (Albany NY). 2021 May 12;13(10):13941–53. doi: 10.18632/aging.203004 (PMC8202848; doi:10.18632/aging.203004)
Supplement: Supplementary Figures [file aging-13-203004-s001.pdf]

SUPPLEMENTARY FIGURES

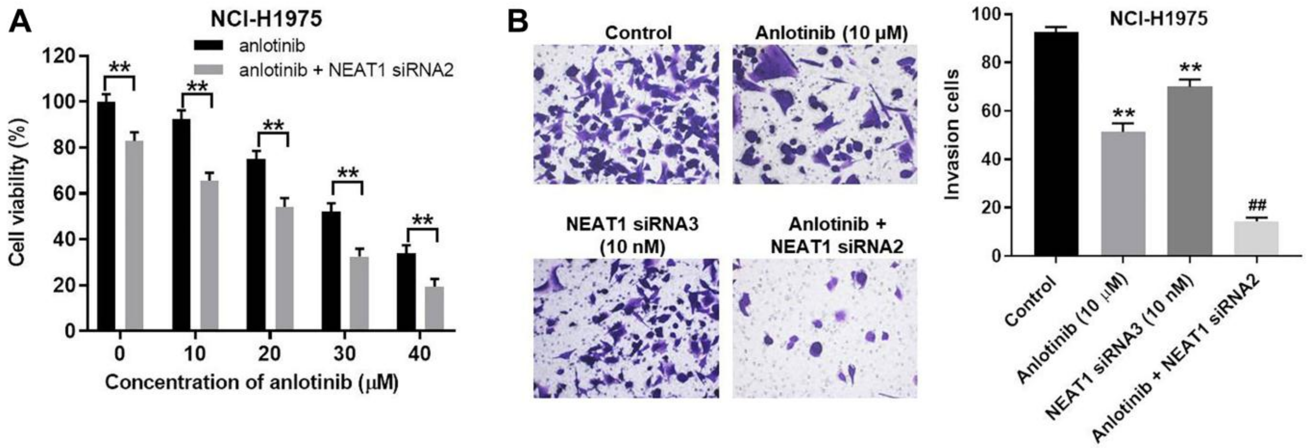

Supplementary Figure 1. NEAT1 knockdown increases the inhibitory effect of anlotinib on NCI-H1975 cell viability and invasion. NCI-H1975 cells were treated with anlotinib or the combination of anlotinib and NEAT1 siRNA 3 for 24 h. (**A**) Cell viability was detected with CCK8 assay. (**B**) Cell invasion was measured with transwell assay. $^{**}P < 0.01$ compared with the control group. $^{\#\#}P < 0.01$, compared with the anlotinib group.

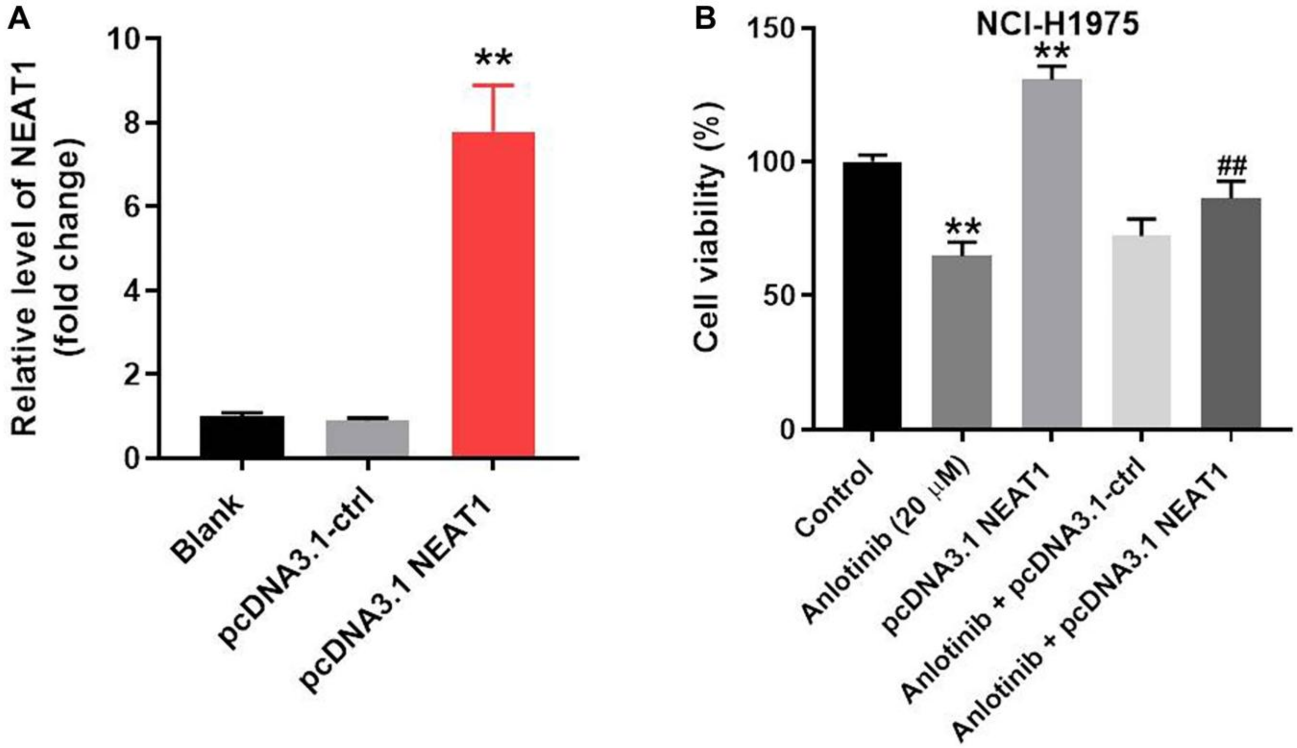

Supplementary Figure 2. Overexpression of NEAT1 reversed the inhibitory effect of anlotinib on NCI-H1975 cell viability. NCI-H1975 cells were transfected with pcDNA3.1 NEAT1 for 24 h. (**A**) The level of NEAT1 in cells was detected with RT-qPCR. (**B**) Cell viability was detected with CCK8 assay. $^{**}P < 0.01$ compared with the control group. $^{\#\#}P < 0.01$, compared with the anlotinib group.

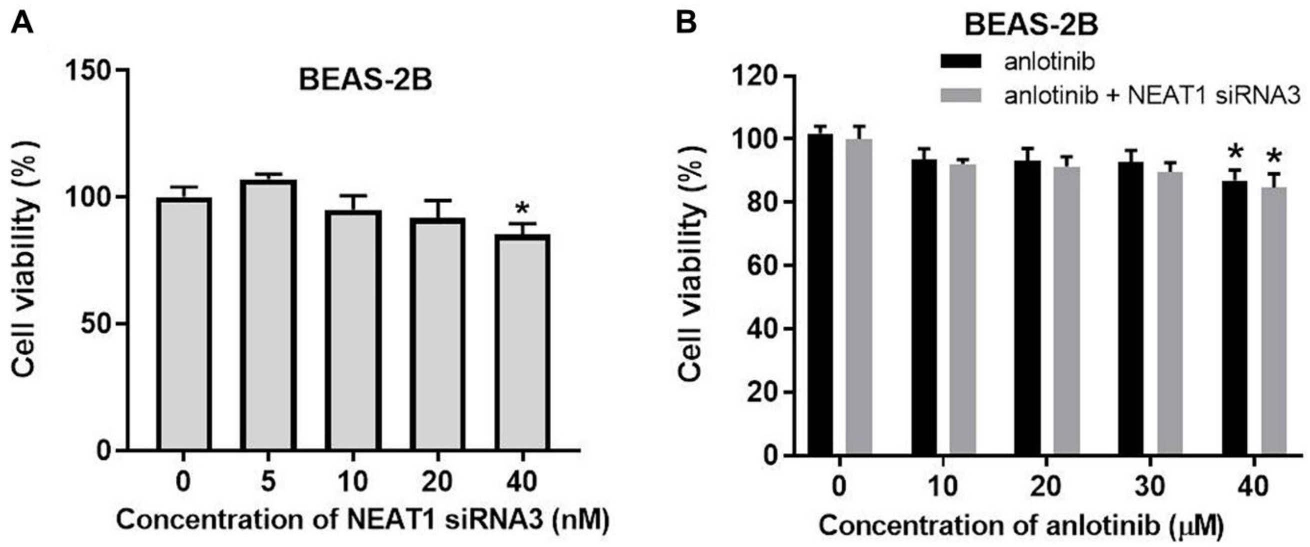

Supplementary Figure 3. The effects of NEAT1 siRNA3 or/and anlotinib on BEAS-2B cell viability. (**A**) BEAS-2B cells were treated with NEAT1 siRNA3 (0, 5, 10, 20, 40 nM) for 24 h; the cell viability was evaluated with CCK8 assays. (**B**) BEAS-2B cells were treated with anlotinib or the combination of anlotinib and NEAT1 siRNA 3 for 24 h. $^{*}P < 0.05$ compared with the control group.
